# TP53 co-mutations in EGFR mutated patients in NSCLC stage IV: A strong predictive factor of ORR, PFS and OS in EGFR mt+ NSCLC

**DOI:** 10.18632/oncotarget.27430

**Published:** 2020-01-21

**Authors:** Julia Roeper, Markus Falk, Athena Chalaris-Rißmann, Anne C. Lueers, Hayat Ramdani, Katrin Wedeken, Ursula Stropiep, Linda Diehl, Markus Tiemann, Lukas C. Heukamp, Fabian Otto-Sobotka, Frank Griesinger

**Affiliations:** ^1^Department of Internal Medicine-Oncology, Carl v. Ossietzky University of Oldenburg, Oldenburg, Germany; ^2^Institute of Hematopathology Hamburg, Hamburg, Germany; ^3^Department of Hematology and Oncology, Pius-Hospital, Oldenburg, Germany; ^4^Department of Tumor Documentation, Cancer Center Oldenburg, Pius Hospital Oldenburg, Oldenburg, Germany; ^5^Division of Epidemiology and Biometry, Carl v. Ossietzky University of Oldenburg, Oldenburg, Germany; ^6^Lung Cancer Network NOWEL.org, Oldenburg, Germany; ^7^Institute of Experimental Immunology and Hepatology, University Medical Center Hamburg-Eppendorf, Hamburg, Germany

**Keywords:** NSCLC, EGFR, TP53 mutation, TKI

## Abstract

Introduction: The impact of TP53 co-mutations in EGFR mutated patients on PFS and OS is controversial. Different classifications of TP53 mutations with respect to functional and potential clinical impact have been published. Therefore, we retrospectively analyzed the impact of TP53 co-mutations on ORR, PFS and OS in a cohort of EGFR mutated NSCLC IV patients (UICC 7) using different classifications of TP53 mutations.

Methods: 75 EGFR mutated NSCLC IV patients homogeneously treated with 1st line EGFR TKI were analyzed for TP53 co-mutations. TP53 mutations were classified according to three different types of classifications. The endpoints ORR, PFS and OS were investigated.

Results: TP53 co-mutations were found in 29/59 patients (49.2%). TP53 co-mutations were a statistically significant independent negative predictive factor for ORR, PFS and OS. TP53 co-mutations were associated with inferior mPFS and mOS: mPFS/mOS 12 vs. 18/24 vs. 42 months for non-disruptive/disruptive mutations vs. WT (*p* < 0.004)/(*p* < 0.009), 11 vs. 17/23 vs. 42 months for pathogenic vs. non-pathogenic/WT (*p* < 0.001)/(*p* < 0.001), and 7 vs. 12 vs. 18/12 vs. 28 vs. 42 months for exon 8 vs. non-exon 8 vs. WT (*p* < 0.001)/(*p* < 0.002).

Conclusions: TP53 co-mutations are frequent in EGFR mt+ NSCLC and have a strong negative impact on all clinical endpoints of TKI therapy.

## INTRODUCTION

EGFR mutations have been identified as one of several driver mutations in NSCLC. EGFR mutations are associated with female sex, non-smoking status and non-squamous histology and occur in the Caucasian NSCLC population with an incidence of 12–15%. EGFR mutations are differentiated into common mutations (exon 19 deletions and exon 21 L858R mutations) and uncommon mutations. The standard of care of EGFR mutated patients is 1st line EGFR TKI treatment based on the results of a number of phase III trials showing a statistically significant advantage of EGFR TKI regarding ORR (objective response rate), PFS (Progression Free Survival), toxicity and quality of life (QoL) as well as in some studies OS (Overall Survival) over chemotherapy [1, 2]. TP53 co-mutations are observed in wild type (WT) NSCLC with an incidence of about 50% and have been associated with smoking status [3].

In EGFR mt+ NSCLC patients the frequency of TP53 mutations ranges from 25.9% [4] and 41% [5] depending on the method of detection. TP53 protein regulates cellular response to stress signals such as chemotherapy, radiation therapy and tyrosine kinase inhibition by inducing cell cycle arrest, senescence and/or apoptosis. Disruption of TP53’s normal function can lead to malignant transformation. Since most chemotherapeutics and most likely TKI’s induce DNA-damage and consequently activate the TP53 protein, mutations in the TP53 gene might negatively affect response to cytotoxic treatment and thus represent a negative predictive factor [8–10]. Also, TP53 mutations might be a negative prognostic factor in lung cancer [11–14], as in other tumor types [15, 16]. However, the impact of TP53 mutations on treatment outcome has not been definitively analysed in EGFR mt+ NSCLC.

Different types of TP53 mutations might confer different prognostic outcome, as shown by Poeta et al. [16]. The autors showed a significantly inferior outcome for EGFRmt+ patients with disruptive TP53 mutations. Classification into disruptive and non-disruptive mutations was based on parameters like localization (i. e. DNA binding domain) and altered biochemical characteristics of the exchanged aminoacid. However, this classification yielded different results in head and neck cancer [16], where disruptive mutations were associated with superior outcomes. An extended functional analysis including additional biophysical parameters (align Grantham Variation Grantham Deviation - GVGD) and a structural analysis might predict more precisely the biologic impact of TP53 mutations [17]. Lastly a third classification that grouped TP53 mutations according to the exon position (exon 8 vs. non-exon 8 mutations) was proposed by Canale et al. [18], where a significant negative impact of exon 8 TP53 mutations on Disease Control Rate (DCR) and PFS in EGFR mt+ NSCLC was shown, however only for the EGFR exon 19 deletion positive cases. No information is available on the stability of TP53 mutations at primary diagnosis and at TKI resistance, and non information as to potential differences in clinical characteristics of EGFR mt+ patients with and without TP53 co-mutations is available.

Therefore, this manuscript addresses the following questions (a) what is the impact of TP53 co-mutations clinically relevant endpoints ORR, PFS and OS in EGFR mt+ positive patients homogeneously treated with a 1st line EGFR TKI (afatinib, erlotinib or gefitinib), (b) do different classifications have different impact on outcome parameters (c) are TP53 mutations associated with distinct clinical characteristics, and (d) is TP53 mutation status stable at acquired TKI resistance.

## RESULTS

### Patients

Overall, 74/75 patients received first line TKI and one patient died before initiation of treatment. First line treatment with erlotinib (33/74; 44.6%), gefitinib (22/74; 29.7%) and afatinib (19/74; 25.7%) and follow-up was performed according to standard guidelines. 69/75 (92%) patients presented with a common mutation, either del19 or L858R in EGFR. 6 patients carried an uncommon EGFR mutation (group I: *n* = 4/III: *n* = 2, according to Yang et al. [19]). The presence of EGFR mutations was associated with female sex (52/75; 69.3%) and never/light smoking status (51/75; 68%). Median age was 66 years. The vast majority of patients presented with an ECOG status 0 (43/75; 57.3%) or 1 (23/75; 30.7%). 38/75 (50.7%) of patients had CCI (Charlson Comorbidity Score) of 2 or 3 points. No dependence as to the type of EGFR mutation was found, as patients with an del19 EGFR mutation had TP53 mutations in 17/42 (40.5%) cases and patients with L858R mutation in 10/27 (37%) cases. For details see appendix (Supplementary Table 1).

### TP53 analysis

**Figure 1:**
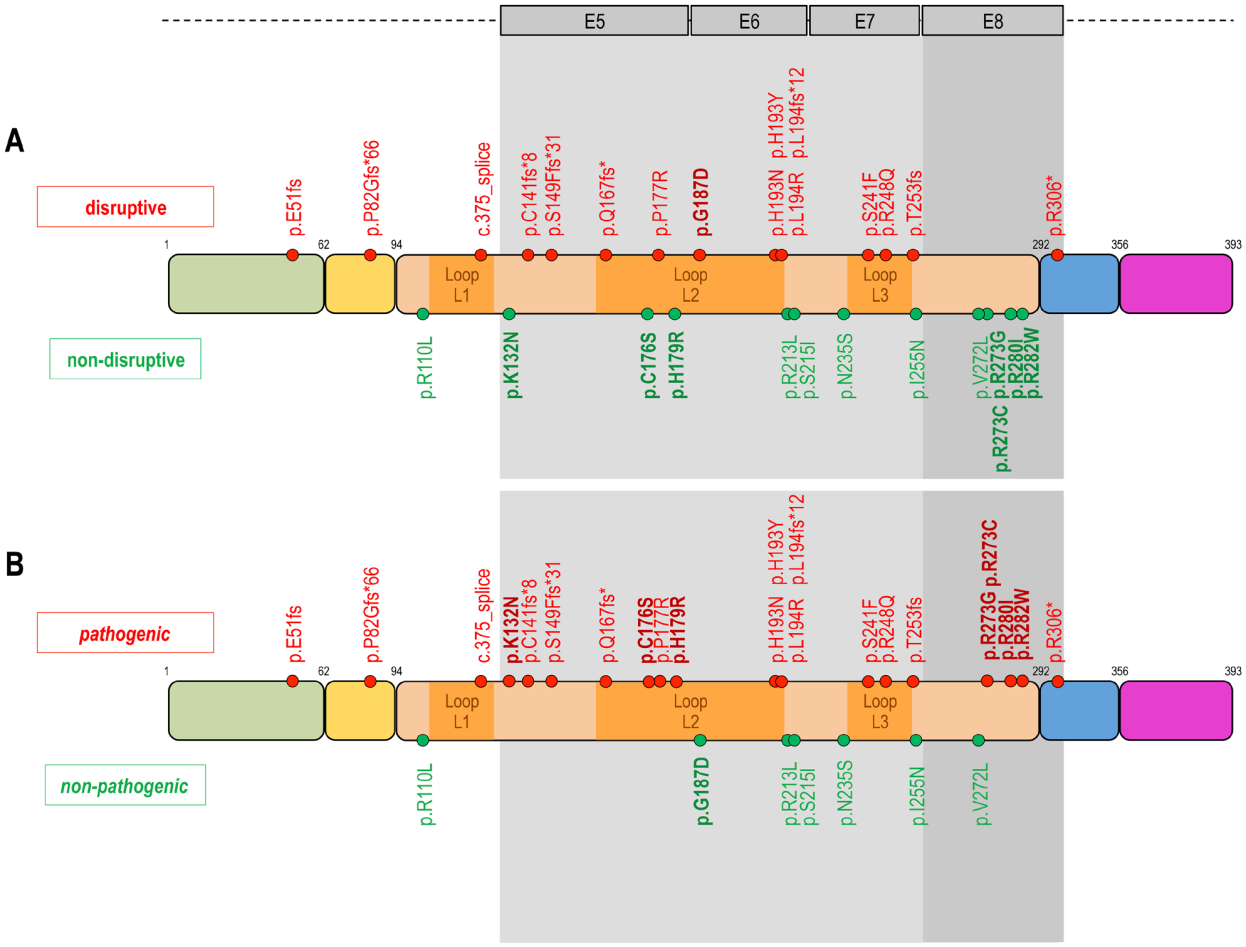
Classification of TP53 mutations. Proposed three different types of classifications of TP53 mutations. The upper DNA strand (**A**) shows the classification according to Poeta et al. 2007, which distinguishes between disruptive (*n* = 16) and non-disruptive mutations (*n* = 13). The disruptive mutations are highlighted in red to illustrate the negative impact on the clinical endpoints (PFS, OS, TKI response). The lower DNA strand (**B**) shows our structural/biophysical classification, which distinguishes between pathogenic and non-pathogenic mutations. The structural/biophysical classification shows more TP53 mutations with a pathogenic (*n* = 22) impact on clinical endpoints. The fields highlighted in grey show the classification according to Canale et al. 2017. Canale et al. grouped TP53 mutations according to the exon position (exon 8 vs. non-exon 8 mutations). TP53 mutations in exon 8 have a more negative impact on clinical endpoints than mutation in non-exon 8. Inspired by Poeta ML, Manola J, Goldwasser MA et al. 2007; Canale M, Petracci E, Delmonte A et al. 2017.

In 59/75 (78.7%) cases TP53 mutation analysis was successful, 16 cases could not be tested because of insufficient tumor material. Figure 1 shows the classification of the different types of TP53 mutations. In 30/59 (50.8%) cases a TP53 WT configuration was observed. TP53 mutations were grouped according to Poeta et al. [16] into non-disruptive (13/59; 22%) and disruptive TP53 mutations (16/59; 27.1%). The structural/biopysical classification resulted in 7/59 (11.9%) patients with non-pathogenic and 22/59 (37.3%) patients with pathogenic TP53 mutation. 6/59 (10.2%) patients had a TP53 exon 8 mutation and 23/59 (39%) a TP53 non-exon 8 mutation.

With the exception of the risk of developing CNS metastases during the course of the disease, no dependence as to any clinical characteristic was observed, including smoking status or type of EGFR mutation. Patients with TP53 mutations (both Poeta et al. [16] and structural/biophysical classifications) developed CNS metastases during the course of the disease in 10/29 (34.5%) cases, in contrast to only 2/29 (6.9%) with TP53 WT (*p* < 0.02 Poeta et al. [16]; *p* < 0.01 structural/biophysical classification). Detailed information is shown in Table 1. Patients with a TP53 mutation have shown a 5 times higher risk of developing CNS metastases during the course of the disease compared to patients with a TP53 WT configuration (OR 5.17 [1.04 to 25.66]; *p* < 0.04). Table 1 shows the clinical characteristics in detail.

**Table 1:** Clinical characteristics of the TP53 patients

TP53 Poeta et al. classification								
	**non-disruptive *n* = 13**	**disruptive *n* = 16**			**TP WT *n* = 30**	**TP53 unknown *n* = 16**	***p*-value **	**ALL *n* = 75**
Age (years)								
**mean**	63 (49–81)	65.5 (50–75)			65.2 (45–85)	67.6 (45–82)	0.72	65.4 (45–85)
**median**	63	67.5			69	67	0.66	66
Sex							0.56	
**men**	4 (30.8%)	7 (43.75%)			7 (23.3%)	5 (31.3%)		23 (30.7%)
**women**	9 (69.2%)	9 (56.25%)			23 (76.7%)	11 (68.7%)		52 (69.3%)
**in total**	13 (100%)	16 (100%)			30 (100%)	16 (100%)		75 (100%)
Smoking status							0.63	
**never/light smoker**	8 (61.5%)	12 (75%)			23 (76.7%)	8 (50%)		51 (68%)
**ex/current smoker**	5 (38.5%)	4 (25%)			7 (23.3%)	5 (31.25%)		21 (28%)
**not known**	0	0			0	3 (18.75%)		3 (4%)
**in total**	13 (100%)	16 (100%)			30 (100%)	16 (100%)		75 (100%)
ECOG							0.56	
**0**	6 (46.1%)	12 (75%)			17 (56.7%)	8 (50%)		43 (57.3%)
**1**	5 (38.5%)	4 (25%)			10 (33.3%)	4 (25%)		23 (30.7%)
**2**	2 (15.4%)	0			3 (10%)	3 (18.8%)		8 (10.7%)
**3**	0	0			0	1 (6.2%)		1 (1.3%)
**in total**	13 (100%)	16 (100%)			30 (100%)	16 (100%)		75 (100%)
CCI							0.48	
**0–1**	7 (53.8%)	6 (37.5%)			8 (26.7%)	3 (18.7%)		24 (32%)
**2–3**	3 (23.1%)	5 (31.25%)			16 (33.3%)	7 (43.8%)		31 (41.3%)
**>3**	3 (23.1%)	5 (31.25%)			6 (20%)	6 (37.5%)		20 (26.7%)
**in total**	13 (100%)	16 (100%)			30 (100%)	16 (100%)		75 (100%)
Exon status							0.94	
**exon 19**	9 (69.2%)	8 (50%)			16 (53.3%)	9 (56.25%)		42 (56%)
**exon 21**	4 (30.8%)	6 (37.5%)			12 (40%)	5 (31.25%)		27 (36%)
**uncommon exons**	0	2 (12.5%)			2 (6.7%)	2 (12.5%)		6 (8%)
**in total**	13 (100%)	16 (100%)			30 (100%)	16 (100%)		75 (100%)
Initial **CNS metastasis CNS primary**							0.57	
• **yes**	3 (23.1%)	3 (18.7%)			9 (30%)	2 (12.5%)		17 (22.7%)
• **no**	10 (76.9%)	13 (81.3%)			21(70%)	14 (87.5%)		58 (77.3%)
**in total**	13 (100%)	16 (100%)			30 (100%)	16 (100%)		75 (100%)
CNS **metastasis PD**							0.02	
• **yes**	6 (46.2%)	4 (25%)			2 (6.7%)	5 (31.3%)		17 (22.7%)
• **no**	7 (53.8%)	12 (75%)			28 (93.3%)	11 (68.7%)		58 (77.3%)
**in total**	13 (100%)	16 (100%)			30 (100%)	16 (100%)		75 (100%)
**TP53 structural/biophysical classification**								
	**non-pathogenic *n* = 7**	**pathogenic *n* = 22**			**TP WT *n* = 30**	**TP53 unknown *n* = 16**	***p*-value **	**All *n* = 75**
Age (years)								
**mean**	67.3 (54–81)	63.5 (49–75)			65.2 (45–85)	67.6 (45–82)	0.65	65.4 (45–85)
**median**	67	63.5			69	67	0.55	66
Sex							0.60	
**men**	2 (28.6%)	9 (40.9%)			7 (23.3%)	5 (31.3%)		23 (30.7%)
**women**	5 (71.4%)	13 (59.1%)			23 (76.7%)	11 (68.7%)		52 (69.3%)
**in total**	7 (100%)	22 (100%)			30 (100%)	16 (100%)		75 (100%)
Smoking status							0.77	
**never/light smoker**	5 (71.4%)	15 (68.2%)			23 (76.7%)	8 (50%)		51 (68%)
**ex/current smoker**	2 (28.6%)	7 (31.8%)			7 (23.3%)	5 (31.25%)		21 (28%)
**not known**	0	0			0	3 (18.75%)		3 (4%)
**in total**	7 (100%)	22 (100%)			30 (100%)	16 (100%)		75 (100%)
ECOG							0.83	
**0**	4 (57.1%)	14 (63.6%)			17 (56.7%)	8 (50%)		43 (57.3%)
**1**	3 (42.9%)	6 (27.3%)			10 (33.3%)	4 (25%)		23 (30.7%)
**2**	0	2 (9.1%)			3 (10%)	3 (18.8%)		8 (10.7%)
**3**	0	0			0	1 (6.2%)		1 (1.3%)
**in total**	7 (100%)	22 (100%)			30 (100%)	16 (100%)		75 (100%)
CCI							0.47	
**0–1**	1 (14.2%)	5 (22.7%)			8 (26.7%)	3 (18.7%)		17 (22.6%)
**2–3**	3 (42.9%)	12 (54.6%)			16 (33.3%)	7 (43.8%)		38 (50.6%)
**>3**	3 (42.9%)	5 (22.7%)			6 (20%)	6 (37.5%)		20 26.6%)
**in total**	7 (100%)	22 (100%)			30 (100%)	16 (100%)		75 (100%)
Exon status							0.95	
e**xon 19**	4 (57.1%)	13 (59.1%)			16 (53.3%)	9 (56.25%)		42 (56%)
e**xon 21**	2 (28.6%)	8 (36.4%)			12 (40%)	5 (31.25%)		27 (36%)
uncommon Exon	1 (14.3%)	1 (4.5%)			2 (6.7%)	2 (12.5%)		6 (8%)
in total	7 (100%)	22 (100%)			30 (100%)	16 (100%)		75 (100%)
Initital CNS metastasis							0.54	
• **yes**	1 (14.3%)	5 (22.7%)			9 (30%)	2 (12.5%)		17 (22.7%)
• **no**	6 (85.7%)	17 (77.3%)			21 (70%)	14 (87.5%)		58 (77.3%)
**in total**	7 (100%)	22 (100%)			30 (100%)	16 (100%)		75 (100%)
CNS metastasis PD							0.01	
• **yes**	4 (57.1%)	6 (27.3%)			2 (6.7%)	5 (31.3%)		17 (22.7%)
• **no**	3 (42.9%)	16 (72.7%)			28 (93.3%)	11 (68.7%)		58 (77.3%)
**in total**	7 (100%)	22 (100%)			30 (100%)	16 (100%)		75 (100%)
**TP53 exon 8 vs. non-exon 8**								
	**TP53 exon 8 *n* = 6**		**TP53 non-exon 8 *n* = 26**	**TP WT *n* = 30**		**TP53 unknown *n* = 16**	***p*-value **	**ALL *n* = 75**
Age (years)								
**mean**	59.3 (49–67)		65.7 (50–81)	65.2 (45–85)		67.6 (45–82)	0.08	65.4 (45–85)
**median**	60		66	69		67	0.35	66
Sex							0.08	
**men**	0		11 (47.8%)	7 (23.3%)		5 (31.3%)		23 (30.7%)
**women**	6 (100%)		12 (52.2%)	23 (76.7%)		11 (68.7%)		52 (69.3%)
**in total**	6 (100%)		23 (100%)	30 (100%)		16 (100%)		75 (100%)
Smoking status							0.60	
**never/light smoker**	5 (83.3%)		15 (65.2%)	23 (76.7%)		8 (50%)		51 (68%)
**ex/current smoker**	1 (16.7%)		8 (34.8%)	7 (23.3%)		5 (31.25%)		21 (28%)
**not known**	0		0	0		3 (18.75%)		3 (4%)
**in total**	6 (100%)		23 (100%)	30 (100%)		16 (100%)		75 (100%)
ECOG							0.83	
**0**	3 (50%)		15 (65.2%)	17 (56.7%)		8 (50%)		43 (57.3%)
**1**	2 (33.3%)		7 (30.4%)	10 (33.3%)		4 (25%)		23 (30.7%)
**2**	1 (16.7%)		1 (4.4%)	3 (10%)		3 (18.8%)		8 (10.7%)
**3**	0		0	0		1 (6.2%)		1 (1.3%)
**in total**	6 (100%)		23 (100%)	30 (100%)		16 (100%)		75 (100%)
CCI							0.27	
**0–1**	3 (50%)		3 (13%)	8 (26.7%)		3 (18.7%)		17 (22.7%)
**2–3**	2 (33.3%)		13 (56.5%)	16 (33.3%)		7 (43.8%)		38 (50.6%)
**>3**	1 (16.7%)		7 (30.5%)	6 (20%)		6 (37.5%)		20 (26.7%)
**in total**	6 (100%)		23 (100%)	30 (100%)		16 (100%)		75 (100%)
Exon status								
**exon 19**	5 (83.3%)		12 (52.2%)	16 (53.3%)		9 (56.25%)		42 (56%)
**exon 21**	1 (16.7%)		9 (39.1%)	12 (40%)		5 (31.25%)		27 (36%)
**uncommon Exon**	0		2 (8.7%)	2 (6.7%)		2 (12.25%)		6 (8%)
**in total**	6 (100%)		23 (100%)	30 (100%)		16 (100%)		75 (100%)
Initial CNS metastasis							0.45	
• **yes**	2 (33.3%)		4 (17.4%)	9 (30%)		2 (12.5%)		17 (22.7%)
• **no**	4 (66.7%)		19 (82.6%)	21 (70%)		14 (87.5%)		58 (77.3%)
**in total**	6 (100%)		23 (100%)	30 (100%)		16 (100%)		75 (100%)
CNS metastasis PD							0.06	
• **yes**	2 (33.3%)		8 (34.8%)	2 (6.7%)		5 (31.3%)		17 (22.7%)
• **no**	4 (66.7%)		15 (65.2%)	28 (93.3%)		11 (68.7%)		58 (77.3%)
**in total**	6 (100%)		23 (100%)	30 (100%)		16 (100%)		75 (100%)

On TKI resistance (1st or 2nd generation TKI) 10/29 (34.5%) patients had a rebiopsy and TP53 status remained stable. In patients with TP53 mutation at the time of primary diagnosis, the same TP53 mutation persisted in the acquired resistance situation in 9/10 (90%) patients, in 1/10 (10%) cases the initial TP53 mutation was not detected most likely due to lack of material. 34/74 (46%) patients were tested for T790M and 20/34 (59%) developed a T790M after 1st line TKI. The risk of developing a T790M+ was independent of the presence of a TP53 mutation at diagnosis. Additional information is shown in the appendix (Supplementary Table 2).

### Objective response rate

ORR was analyzed using the three TP53 classifications in 72 patients (1 died prior to start of TKI, 2 died prior of first assessment of response). The CR/PR rate in TP53 WT patients was 27/28 (96.4%) and 20/29 (69%) for non-disruptive/disruptive TP53 mutations. The odds to reach CR/PR was 12.15 for patients with WT vs. non-disruptive/disruptive TP53 mutation (*p* < 0.02). 15/22 (68.2%) of patients with a pathogenic and 5/7 (71.4%) of patients with a non-pathogenic TP53 mutation achieved a CR/PR. The odds to reach CR/PR was 4.98 for TP53 WT/non-pathogenic mutations vs. pathogenic TP53 mutations (*p* < 0.03). 4/6 (66.7%) of patients with an exon 8 mutation and 16/23 (69.6%) of patients with a non-exon 8 mutation had a CR/PR. The odds to reach CR/PR was 13.5 for patients with TP53 WT vs. exon 8 mutations (*p* < 0.05). Thus TP53 co-mutations are a strong negative predictor of ORR. Additional information is shown in Table 2.

**Table 2:** Objective response rate

**ORR in percent % (*n*)**						
**EGFR exon status**						
			**exon 19**	**exon 21**	**uncommon EGFR mt+**	
**ORR**	CR/PR		92.9% (39)	76% [19]	40% [2]	
	SD/PD		7.1% [3]	24% [6]	60% [3]	
	total		100% (42)	100% [25]	100% [5]	
			**Odds Ratio CR/PR**		***p*-value **	
**exon 19 vs. Exon 21**			4.11 [0.92–18.22]		0.06	
exon 19 vs. uncommon EGFR mt+			19.50 [2.29–165.76]		0.007	
exon 21 vs. uncommon EGFR mt+			4.75 [0.64–35.48]		0.13	
**EGFR exon 19/21 and TP53 mutation status**						
			**exon 19/TP53 mt+**	**exon 19/TP53 WT**	**exon 21/TP53 mt+**	**exon 21/TP53 WT**
**ORR**	CR/PR		77.8% [14]	100% [16]	60% [6]	90.9% [10]
	SD/PD		22.2% [4]	0	40% [4]	9.1% [1]
	total		100% [18]	100% [16]	100% [10]	100% [11]
			**Odds Ratio CR/PR**		***p*-value **	
**exon 19/TP53 WT vs. exon 19/TP53 mt+**			10.24 [0.51–206.88]		0.13	
**exon 21/TP53 WT vs. exon 21/TP53 mt+**			6.67 [0.60–74.51]		0.12	
**exon 19/TP53 mt+ vs. exon 21 TP53 mt+**			2.33 [0.42–12.57]		0.32	
**TP53 mutation and T790M+/**-						
			**TP53 mt/T790M+**		**TP53 WT/T790M+**	
**ORR**		CR/PR	100% [10]		100% [9]	
		SD/PD	0		0	
		total	100% [10]		100% [9]	
			**Odds Ratio CR/PR**		***p*-value **	
**TP53 mt/T790M+ vs. TP53WT/T790M+**			1		1	
**TP53 Poeta et al. classification**						
			**non-disruptive**	**disruptive**	**TP53 WT**	**TP53 unknown**
**ORR**	CR/PR		61.5% [8]	75% [12]	96.4% [27]	86.7% [13]
	SD/PD		38.5% [5]	25% [4]	3.6% [1]	13.3% [2]
	total		100% [13]	100% [16]	100% [28]	100% [15]
			**Odds Ratio CR/PR**		***p*-value **	
**disruptive vs. non-disruptive**			1.88 [0.38–9.20]		0.44	
**TP53 WT vs. non-disruptive**			16.88 [1.71–166.21]		0.02	
**TP53 WT vs. disruptive**			9.00 [0.91–89.27]		0.06	
**TP53 WT vs. non-disruptive/disruptive**			12.15 [1.42–103.82]		0.02	
**TP53 Structural/biophysical classification**						
			**non-pathogenic**	**pathogenic**	**TP53 WT**	**TP53 unknown**
**ORR**	CR/PR		71.4% [5]	68.2% [15]	96.4% [27]	86.7% [13]
	SD/PD		28.6% [2]	31.8% [7]	3.6% [1]	13.3% [2]
	total		100% [7]	100% [22]	100% [28]	100% [15]
			**Odds Ratio CR/PR**		***p*-value **	
**non-pathogenic vs. pathogenic**			1.17 [0.18–75.6]		0.87	
TP53 WT vs. non-pathogenic			10.80 [0.82–142.98]		0.07	
**TP53 WT vs. pathogenic**			12.60 [1.41–112.39]		0.02	
**TP53 WT/non-pathogenic vs. pathogenic**			4.98 [1.13–21.98]		0.03	
**TP53 exon 8 vs. non-exon 8 classification**						
			**Exon 8**	**other Exons**	**TP53 WT**	**TP53 unknown**
**ORR**	CR/PR		66.7% [4]	69.6% [16]	96.4% [27]	86.7% [13]
	SD/PD		33.3% [2]	30.4% [7]	3.6% [1]	13.3% [2]
	total		100% [6]	100% [23]	100% [28]	100% [15]
			**OR CR/PR**		***p*-value **	
**non-Exon 8 vs. Exon 8**			1.14 [1.33–104.98]		0.89	
**TP53 WT vs. Exon 8**			13.50 [0.98–185.45]		0.05	
**TP53 WT vs. non-exon 8**			11.81 [1.33–104.48]		0.03	

In the multivariate analysis TP53 non-disruptive/disruptive and TP53 pathogenic mutations remained independent negative predictive factors for ORR: Other independent factors are EGFR uncommon mutations, ECOG status 2 and the variable no initial CNS metastases. The complete results are shown in the appendix (Supplementary Table 3).

### Progression free survival

**Figure 2:**
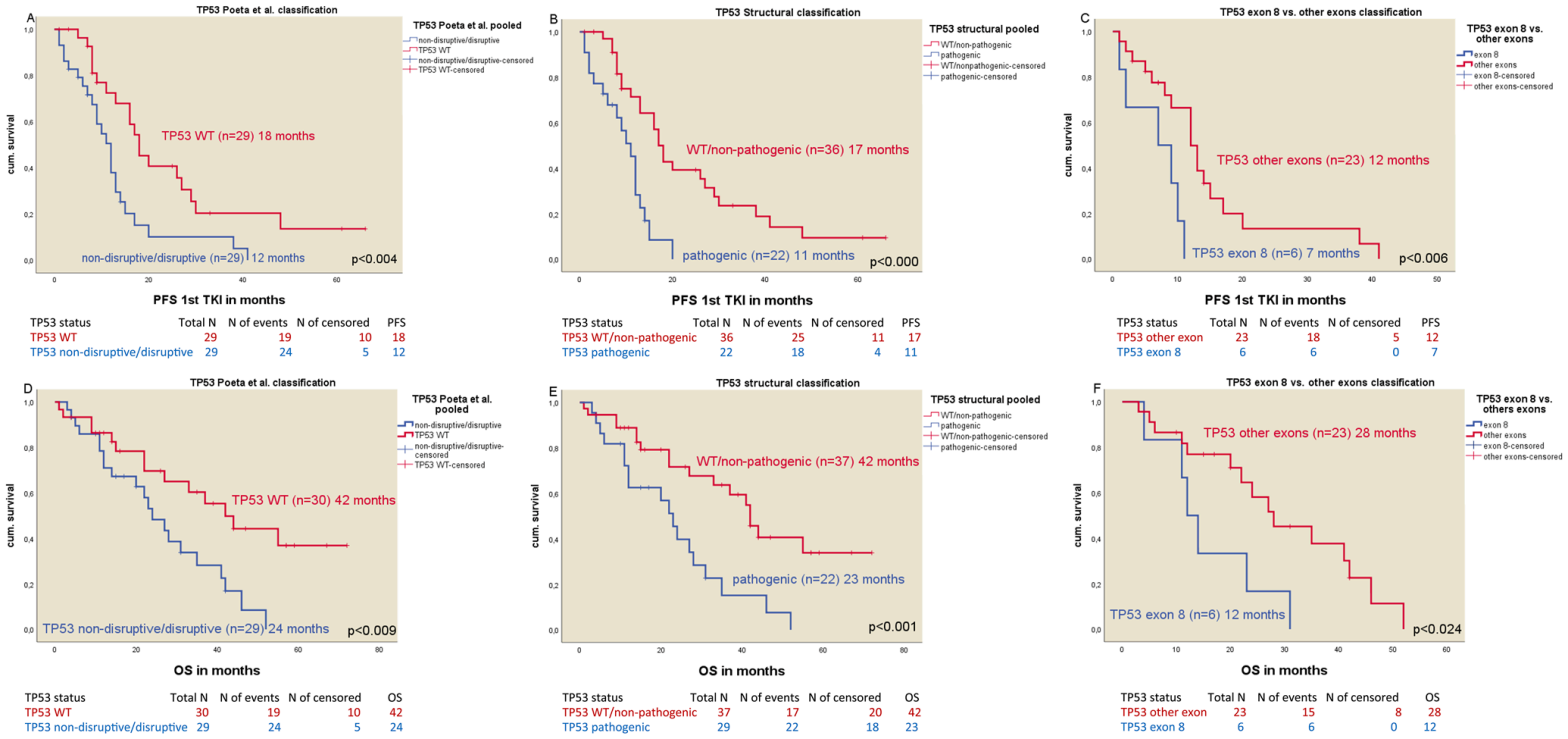
Kaplan-Meier plots of the different classification of TP53 mutations. (**A**) Median PFS of TP53 Poeta et al. non-disruptive/disruptive mt+ vs. TP53 WT. (**B**) Median PFS of TP53 structural/biopysical classification TP53 non-pathogenic/TP53 WT vs. TP53 pathigenic mt+. (**C**) Median PFS of TP53 exon 8 vs. TP53 non-exon 8 mt+. (**D**) Median OS of TP53 Poeta et al. non-disruptive/disruptive mt+ vs. TP53 WT. (**E**) Median OS of TP53 structural/biophysical classification TP53 non-pathogenic/TP53 WT vs. TP53 pathogenic mt+. (**F**) Median OS of TP53 exon 8 vs. TP53 non-exon 8 mt+.

Statistically significant differences in median PFS were found in all TP53 mutation classifications (Table 3): mPFS in non-disruptive/disruptive TP53 mutations (*n* = 29) vs. WT TP53 mutations (*n* = 29) was 12 vs. 18 months, respectively (*p* < 0.004) (Figure 2A) (HR 0.41; *p* < 0.005). The median PFS of TP53 WT/non-pathogenic mutations (*n* = 36) was significantly different to pathogenic TP53 mutated patients (*n* = 22) with 17 months vs. 11 months respectively (*p* < 0.001) (Figure 2B) (HR 0.28; *p* < 0.001). Patients with a TP53 mutation in exon 8 (*n* = 6) had a median PFS of 7 months vs. 12 months for patients with non-exon 8 TP53 mutations (*n* = 23) (*p* < 0.006) (Figure 2C) (HR 0.15; *p* < 0.001). The negative impact of TP53 co-mutations for mPFS was seen in both del19 and exon21 mutated patients (Table 3).

**Table 3:** Median PFS in months

**Median PFS in months**				
**EGFR exon status**				
	**Median PFS**	**95% CI**	***n***	***p*-value **
**EGFR exon 19**	13	10.228–15.772	42	
**EGFR exon 21**	16	11.983–20.017	27	
**EGFR uncommon mutation**	9	4.022–13.978	5	
				**0.792**
	**Median PFS**	**95% CI**	***n***	***p*-value **
**EGFR exon 19/TP53 mt+**	11	8.359–13.641	18	
**EGFR exon 21/TP53 WT**	20	12.351–27.649	16	
				**<0.001**
	**Median PFS**	**95% CI**	***n***	***p*-value **
**EGFR exon 21/TP53 mt+**	10	4.300–15.700	10	
**EGFR exon 21/TP53 WT**	17	8.161–25.835	12	
				**0.453**
**TP53 mutation and T790M+/**-				
	**Median PFS**	**95% CI**	***n***	***p*-value **
**TP53/T790M+**	7	5.379–8.621	10	
**TP53 WT/T790M+**	10	2.679–17.321	9	
				**0.23**
**TP53 Poeta et al. classification**				
	**Median PFS**	**95% CI**	***n***	***p*-value **
**non-disruptive**	10	6.721–13.279	13	
**disruptive**	12	7.633–16.367	16	
**TP53 WT**	18	13.5–22.5	29	
**TP53 unknown**	15	6.909–23.091	16	
				**0.18**
	**Median PFS**	**95% CI**	***n***	***p*-value **
**non-disruptive**	10	6.721–13.279	13	
**disruptive**	12	7.633–16.367	16	
**TP53 WT**	18	13.5–22.5	29	
				**0.007**
	**Median PFS**	**95% CI**	***n***	***p*-value **
**non-disruptive**	10	6.721–13.279	13	
**disruptive**	12	7.633–16.367	16	
				**0.224**
	**Median PFS**	**95% CI**	***n***	***p*-value **
**non-disruptive**	10	6.721–13.279	13	
**TP53 WT**	18	13.5–22.5	29	
				**0.004**
	**Median PFS**	**95% CI**	***n***	***p*-value **
**disruptive**	12	7.633–16.367	16	
**TP53 WT**	18	13.5–22.5	29	
				**0.033**
	**Median PFS**	**95% CI**	***n***	***p*-value **
**non-disruptive/disruptive**	12	9.271–14.729	29	
**TP53 WT**	18	13.5–22.5	29	
				**0.004**
**TP53 structural/biophysical classification**				
	**Median PFS**	**95% CI**	***n***	***p*-value **
**non-pathogenic**	13	3.398–22.602	7	
**pathogenic**	11	8.63–13–37	22	
**TP53 WT**	18	13.5–22.5	29	
**TP53 unknown**	15	6.909–23.091	16	
				**0.001**
	**Median PFS**	**95% CI**	***n***	***p*-value **
**non-pathogenic**	13	3.398–22.602	7	
**pathogenic**	11	8.63–13–37	22	
**TP53 WT**	18	13.5–22.5	29	
				**0.001**
	**Median PFS**	**95% CI**	***n***	***p*-value **
**non-pathogenic**	13	3.398–22.602	7	
**TP53 WT**	18	13.5–22.5	29	
				**0.542**
	**Median PFS**	**95% CI**	***n***	***p*-value **
**pathogenic**	11	8.63–13–37	22	
**TP53 WT**	18	13.5–22.5	29	
				**<0.001**
	**Median PFS**	**95% CI**	***n***	***p*-value **
**non-pathogenic**	13	3.398–22.602	7	
**pathogenic**	11	8.63–13–37	22	
				**0.059**
	**Median PFS**	**95% CI**	***n***	***p*-value **
TP53 WT/non-pathogenic	17	14.458–19.541	36	
**pathogenic**	11	8.63–13–37	22	
				**<0.001**
**TP53 exon 8 vs. non-exon 8 classification**				
	**Median PFS**	**95% CI**	***n***	***p*-value **
**exon 8**	7	0.000–15.402	6	
**non-exon 8**	12	8,757–15.243	23	
**TP53 WT**	18	13.5–22.5	29	
**TP53 unknown**	15	6.909–23.091	16	
				**<0.001**
	**Median PFS**	**95% CI**	***n***	***p*-value **
**exon 8**	7	0.0–15.402	6	
**non-exon 8**	12	8.757–15.243	23	
**TP53 WT**	18	13.5–22.5	29	
				**<0.001**
	**Median PFS**	**95% CI**	***n***	***p*-value **
**exon 8**	7	0.0–15.402	6	
**non-exon 8**	12	8.757–15.243	23	
				**0.006**
	**Median PFS**	**95% CI**	***n***	***p*-value **
**exon 8**	7	0.0–15.402	6	
**TP53 WT**	18	13.5–22.5	29	
				**<0.001**
	**Median PFS**	**95% CI**	***n***	***p*-value **
**non-exon 8**	12	8.757–15.243	23	
**TP53 WT**	18	13.5–22.5	29	
				**0.024**

In the multivariate analysis, TP53 non-disruptive/disruptive mutations, TP53 pathogenic mutation, ECOG status 2 and the EGFR mutation status were independent predictive factors for PFS. A shorter median PFS is associated with TP53 non-disruptive/disruptive mutations vs. TP53 WT (HR 3.07; *p* < 0.003) and with TP53 pathogenic mutations vs. TP53 WT/non-pathogenic mutations (HR 6.19; *p* < 0.001). The full results are shown in the appendix (Supplementary Table 3).

### Overall survival

mOS was different in EGFR mt+ patients based on the TP53 mutation status and the different classifications (Table 4). The median OS of patients with non-disruptive/disruptive TP53 mutation (*n* = 29) was 24 months compared to 42 months in patients with TP53 WT (*n* = 30, *p* < 0.009, Figure 2D) (HR 0.40; *p* < 0.012) and 42 months in TP53 WT/non-pathogenic mutations (*n* = 37) vs. 23 in patients with pathogenic TP53 mutations (*n* = 22, *p* < 0.001, Figure 2E) (HR 0.33; *p* < 0.002). The median OS in patients with TP53 exon 8 mutations (*n* = 6) was 12 months vs. 28 months in patients with a non-exon 8 TP53 mutation (*n* = 23, *p* < 0.024, Figure 2F) vs. TP53 WT (42 months) (HR 0.21; *p* < 0.004). TP53 mutations were an independent predictive factor in both del19 and exon 21 mutated patients for median OS. Detailed information is shown in Table 4.

**Table 4:** Median OS in months

Median OS in months				
**EGFR exon status**				
	**Median OS**	**95% CI**	***n***	***p*-value **
**EGFR exon 19**	31	24.904–37.096	42	
**EGFR exon 21**	20	12.943–27.057	27	
**EGFR uncommon mutation**	8	0.000–46.408	6	
				**0.166**
	**Median OS**	**95% CI**	***n***	***p*-value **
**EGFR exon 19/TP53 mt+**	27	20.266–33.734	18	
**EGFR exon 19/TP53 WT**	55	**-**	16	
				**0.005**
	**Median OS**	**95% CI**	***n***	***p*-value **
**EGFR exon 21/TP53 mt+**	11	0.000–22.833	10	
**EGFR exon 21/TP53 WT**	22	0.000–47.183	12	
				**0.09**
**TP53 mutation and T790M+/**-				
	**Median OS**	**95% CI**	***n***	***p*-value **
**TP53/T790M+**	35	17.915–52.081	10	
**TP53 WT/T790M+**	55	34.295–75.705	9	
				**0.094**
**TP53 Poeta et al. classification**				
	**Median OS**	**95% CI**	***n***	***p*-value **
**non-disruptive**	27	11.065–42.935	13	
**disruptive**	24	17.040–30.960	16	
**TP53 WT**	42	28.754–55–246	30	
**TP53 unknown**	15	7.160–22.840	16	
				**0.010**
	**Median OS**	**95% CI**	***n***	***p*-value **
**non-disruptive**	27	11.065–42.935	13	
**disruptive**	24	17.040–30.960	16	
**TP53 WT**	42	28.754–55–246	30	
				**0.032**
	**Median OS**	**95% CI**	***n***	***p*-value **
**non-disruptive**	27	11.065–42.935	13	
**disruptive**	24	17.040–30.960	16	
				**0.862**
	**Median OS**	**95% CI**	***n***	***p*-value **
**non-disruptive**	27	11.065–42.935	13	
**TP53 WT**	42	28.754–55–246	30	
				**0.077**
	**Median OS**	**95% CI**	***n***	***p*-value **
**disruptive**	24	17.040–30.960	16	
**TP53 WT**	42	28.754–55–246	30	
				**0.018**
	**Median OS**	**95% CI**	***n***	***p*-value **
**non-disruptive/disruptive**	24	17.019–30.981	29	
**TP53 WT**	42	28.754–55–246	30	
				**0.009**
**TP53 structural/biophysical classification**				
	**Median OS**	**95% CI**	***n***	***p*-value **
**non-pathogenic**	42	40.360–43.640	7	
**pathogenic**	23	17.782–28.218	22	
**TP53 WT**	42	28.754–55–246	30	
**TP53 unknown**	15	7.160–22.840	16	
				**0.002**
	**Median OS**	**95% CI**	***n***	***p*-value **
**non-pathogenic**	42	40.360–43.640	7	
**pathogenic**	23	17.782–28.218	22	
**TP53 WT**	42	28.754–55–246	30	
				**0.005**
	**Median OS**	**95% CI**	***n***	***p*-value **
**non-pathogenic**	42	40.360–43.640	7	
**TP53 WT**	42	28.754–55–246	30	
				**0.876**
	**Median OS**	**95% CI**	***n***	***p*-value **
**pathogenic**	23	17.782–28.218	22	
**TP53 WT**	42	28.754–55–246	30	
				**0.003**
	**Median OS**	**95% CI**	***n***	***p*-value **
**non-pathogenic**	42	40.360–43.640	7	
**pathogenic**	23	17.782–28.218	22	
				**0.127**
	**Median OS**	**95% CI**	***n***	***p*-value **
WT/non-pathogenic TP53	42	34.711–49.289	37	
**pathogenic**	23	17.782–28.218	22	
				**0.001**
**TP53 exon 8 vs. non-exon 8 classification**				
	**Median OS**	**95% CI**	***n***	***p*-value **
**exon 8**	12	8.399–15.601	6	
**non-exon 8**	28	15.168–40.184	23	
**TP53 WT**	42	28.754–55–246	30	
**TP53 unknown**	15	7.160–22.840	16	
				**0.001**
	**Median OS**	**95% CI**	***n***	***p*-value **
**exon 8**	12	8.399–15.601	6	
**non-exon 8**	28	15.168–40.184	23	
**TP53 WT**	42	28.754–55–246	30	
				**0.002**
	**Median OS**	**95% CI**	***n***	***p*-value **
**exon 8**	12	8.399–15.601	6	
**non-exon 8**	28	15.168–40.184	23	
				**0.024**
	**Median OS**	**95% CI**	***n***	***p*-value **
**exon 8**	12	8.399–15.601	6	
**TP53 WT**	42	28.754–55–246	30	
				**0.001**
	**Median OS**	**95% CI**	***n***	***p*-value **
**non-exon 8**	28	15.168–40.184	23	
**TP53 WT**	42	28.754–55.246	30	
				**0.051**

In the multivariate analysis for OS including the covariates TP53 non-disruptive/disruptive mutation, ECOG status 2, type of EGFR mutation, TP53 pathogenic mutation and no initial CNS metastasis, TP53 mutations, only non-disruptive/disruptive and pathogenic TP53 mutations remained as independent factors for survival: HR 4.08, *p* < 0.001 and HR 4.88, *p* < 0.001, respectively. Additional information is shown in the appendix (Supplementary Table 3).

## DISCUSSION

This paper extensively studies the impact of TP53 co-mutations in EGFR mutated NSCLC homogeneously treated with EGFR TKI in first line on clinically relevant endpoints ORR, PFS and OS. In our study of 75 patients with EGFR mt+ tumors, the incidence of TP53 mutations was 29/59 patients (49%) and thus more frequent than previously reported using Sanger sequencing yielding 26% [4] and 30% [18], however consistent with more sensitive methods used by VanderLaan et al. (TP53 incidence 50%) [20] and Labbé et al. (41%) [5], the Cologne group (48%) [21] and up to 62% as observed by Yu et al. [22] using hybrid capture methodology.

Although TP53 mutations have been associated with exogenous toxins such as smoking, no dependence with to the smoking status categorizing patients in never/light and heavy smokers in our cohort was observed. Also, TP53 mutations were not associated with any other prognostically significant factors such as age, ECOG, CNS metastases at diagnosis or type of EGFR mutation. The high frequency of TP53 mutations seems to be a hallmark of EGFR mutated NSCLC, as they are much less frequent in ALK+ tumors, another tumor predominantly observed in never or light smokers. Furthermore, TP53 mutations seem to be a stable genetic marker in EGFR mt+ NSCLC as no conversions of the TP53 status were observed at the time point of acquired TKI resistance. This is also in contrast to ALK+ tumors, where TP53 mutations respresent on of multiple resistance mechanisms of ALK inhibitors [23].

In this retrospective analysis of EGFR mutated patients, a significant impact of TP53 mutations on ORR, PFS and OS was found. This unfavorable impact was independent of the type of EGFR mutation (del19 vs. exon 21), and in a multivariate analysis TP53 mutations represented an independent unfavorable predictor for ORR, PFS and OS. The hypothesis that TP53 mutations are associated with a lower incidence of T790M mutations could be discarded in the cohort described. 10/29 (34.5%) patients with a TP53 mutation and 9/29 (31%) patients with a TP53 WT developed a T790M resistance mutation after 1st line TKI. When patients with acquired resistance were rebiopsied, the incidence of T790M, which is associated with a more favorable outcome than non T790M resistance mutations [24], there was no significant difference in the incidence of TP53 mutations. Also when patients with and without T790M were analyzed, TP53 mutations still had a significant impact on PFS and OS.

One potential explanation for the unfavorable outcome of the TP53 mutated patients might be the risk of developing CNS metastases during the course of the disease: while the incidence of CNS metastases was not different in the TP53 WT vs. TP53 mt+ cohorts at diagnosis, the risk of developing CNS metastases during the course of the disease was higher in the TP53 mutation subgroup than in the TP53 WT cohort (10/29; (34.4%) of TP53 mt+ patients vs. 2/29; (6.9%) of TP53 WT patients; *p* < 0.02; *p* < 0.01). Thus it might be speculated that TP53 mutations are associated with a higher risk of seeding in the brain. In ALK+ NSCLC MRI of the brain are performed every 3 to 6 months due the high incidence of brain metastastases. In contrast there is no widely accepted follow up regimen in EGFR mt+ NSCLC regarding brain imaging. The data presented for EGFR mutated and TP53 co-mutated NSCLC patients might stipulate for a systematic and regular MRI imaging of the brain as part of the follow up of these patients at high risk for developing brain metastases.

In the current paper, we compared different classifications of TP53 mutations that were used in the literature. Overall, the unfavorable impact on clinical outcome parameters was seen for all TP53 mutations, however the “structural/biophysical classification” and the classification using TP53 exon 8 mutations [17] correlated more precisely with outcome parameters than the classification based on Poeta et al. [16] and used by the Molina-Vila et al. [4]. However, we cannot formally distinguish between p53 being a predictive or a prognostic marker because in our study we do not have data on EGFR mt+ patients that were untreated or that received chemotherapy instead of TKI. Prognostic factors are defined as factors independent of treatment and predictive factors as those dependent of treatment and predictive of the the effectiveness of treatments [25]. Therefore, as we included only patients treated with TKI in our study, we opted for defining TP53 mutations as being predictive for the effectiveness of TKI treatment. To definitively answer the question of TP53 being a predictive or prognostic marker, a multicenter retrospective study that compares the outcome with and without TKI therapy of TP53 comutations would be necessary.

Recently the combination of chemotherapy and EGFR TKI was studied in a prospective phase III trial that yielded a significant OS benefit for the combination therapy [26]. It might be speculated that TP53 mutated tumors might preferentially benefit from a broad cytotoxic chemotherapeutic approach in combination with EGFR TKI. A stratification for TP53 mutations should at least be performed in the upcoming EGFR TKI and chemotherapy trials.

An even more attractive approach to tackle TP53 mutated EGFR mt+ tumors might be the specific targeting of TP53 mutations by TP53 inhibitors. This approach is being discussed extensively in a variety of cancers, such as breast cancer [27] and hematologic malignancies [28]. Recently a trial combining a TP53 inhibitor (APREA 246) in combination with azacitidine in TP53 mutant MDS showed a 100% response rate with 8/9 patients even achieving complete remission with median PFS and median OS not reached. As to the knowledge of the authors trials combining TP53 targeted therapies and EGFR TKI have not been initiated in EGFR mt+ NSCLC but this patient population could potentially represent a good target for such a specific approach.

## MATERIALS AND METHODS

Between 2009 and 2016 465 patients from a single center (Pius-Hospital Oldenburg, Germany) diagnosed with NSCLC IV were studied for the presence of EGFR mutations. In routine diagnostic patients were prospectively tested for EGFR and other biomarker.

### Patients

75/465 (16%) patients with TTF1 positve adenocarcinoma of the lung ICD-O M8140/3 harboring sensitizing EGFR mutations and intended for 1st line EGFR TKI treatment were included in this retrospective analysis and were further analyzed for the presence of TP53 mutations. Two cycles of 1st line chemotherapy were allowed because of turn around time of the EGFR results. Patients’ clinical and molecular data including sex, age, histology, smoking status, ECOG, presence of CNS metastases at baseline and during course of disease and comorbidities, as well as types of EGFR and TP53 mutations were captured. ORR, PFS and OS were assessed. Patients gave informed and written consent and the project was approved by the University of Oldenburg’s ethics committee (FP-Projekt 2014-I).

### TP53 co-mutation

Mutational analyses were performed by standard methods, mostly by hybrid capture assays. DNA was extracted from FFPE tissue and subjected to molecular analysis for EGFR, TP53 and other therapeutically relevant genes. TP53 mutations were classified according to different algorithms 1: as previously described by Poeta et al. [16], 2: by an extended algorithm based on Poeta et al. [16] with additional parameters like structural prediction and GVGD biophysical analysis [17] and 3: based on exon 8 vs. non-exon 8 mutations [18]. More details are given in the appendix (Supplementary Materials - Patients and Methods).

### Statistics

For this retrospective analysis, bivariate dependencies with OS and PFS were using Kaplan Meier curves and the log rank test for the calculation of *p*-values. For ORR a logistic regression considering the covariates (sex, age, ECOG, smoking status, EGFR exon status, CNS metastasis and the CCI) was used. The logistic regression results are reported as odds ratios with 95% confidence intervals. Furthermore we constructed Cox regression models to predict the PFS and the OS separately in multivariate analyses. The results are presented as hazard ratios with 95% confidence intervals and *p*-values.

## SUPPLEMENTARY MATERIALS



## Abbreviations

APREA: APREA therapeutics
CCI: Charlson Comorbidity Index
CI: Confidence Interval
CNS: Central Nervous System
CR/PR: Complete Response/Partial response
DCR: Disease Control Rate
del19: Deletion 19
DFG: Deutsche Forschungsgemeinschaft
DNA: Deoxyribonucleic Acid
ECOG: Eastern Cooperative Oncology Group
EGFR: Epidermal Growth Factor-Receptor
FFPE: Formalin-Fixed, Paraffin-Embedded
FP: Forschungspool
GVGD: Grantham Variation Grantham Deviation
HR: Hazard Ratio
i.e.: id est
MDS: Myelodysplastic Syndromes
MRI: Magnetic Resonance Imaging
mt+: mutated
NSCLC: Non-Small-Cell Lung Cancer
OR: Odds Ratio
ORR: Objective Response Rate
OS: Overall Survival
PFS: Progression Free Survival
pts: patients
Qol: Quality of Life
TKI: Tyrosinkinase-Inhibitor
TP53: Tumor suppressor gene (p53)
UICC: Union for International Cancer Control
vs.: versus
WT: Wild Type

## References

[1] Lee CK , Davies L , Wu YL , Mitsudomi T , Inoue A , Rosell R , Zhou C , Nakagawa K , Thongprasert S , Fukuoka M , Lord S , Marschner I , Tu YK , et al. Gefitinib or Erlotinib vs chemotherapy for EGFR mutation-positive lung cancer: Individual patient data meta-analysis of overall survival. J Natl Cancer Inst. 2017; 1:109. 10.1093/jnci/djw279. 28376144

[2] Roeper J , Griesinger F . Epidermal growth factor receptor tyrosine kinase inhibitors in advanced nonsmall cell lung cancer: what is the preferred first-line therapy? Curr Opin Oncol. 2019; 31:1–7. 10.1097/CCO.0000000000000495. 30451714

[3] Jincui G , Yanbin Z , Lixia H , Weijun O , Jian W , Shaoli L , Junwen X , Jinlun F , Baomo L . TP53 mutation is associated with a poor clinical outcome for non-small cell lung cancer: Evidence from a meta-analysis. Mol Clin Oncol. 2016; 5:705–713. 10.3892/mco.2016.1057. 28101350 PMC5228103

[4] Molina-Vila MA , Bertran-Alamillo J , Gascó A , Mayo-de-las-Casas C , Sánchez-Ronco M , Pujantell-Pastor L , Bonanno L , Favaretto AG , Cardona AF , Vergnenègre A , Majem M , Massuti B , Morán T , et al. Nondisruptive p53 mutations are associated with shorter survival in patients with advanced non-small cell lung cancer. Clin Cancer Res. 2014; 20:4647–4659. 10.1158/1078-0432.CCR-13-2391. 24696321

[5] Labbé C , Cabanero M , Korpanty GJ . Prognostic and predictive effects of TP53 co-mutation in patients with EGFR-mutated non-small cell lung cancer (NSCLC). Lung Cancer. 2017; 111:23–29. 10.1016/j.lungcan.2017.06.014. 28838393

[6] Zilfou JT , Lowe SW . Tumor suppressive functions of p53. Cold Spring Harb Perspect Biol. 2009; 1:a001883. 10.1101/cshperspect.a001883. 20066118 PMC2773645

[7] Chène P . The role of tetramerization in p53 function. Oncogene. 2001; 20:2611–7. 10.1038/sj.onc.1204373. 11420672

[8] Viktorsson K , De Petris L , Lewensohn R . The role of p53 in treatment responses of lung cancer. Biochem Biophys Res Commun. 2005; 331:868–80. 10.1016/j.bbrc.2005.03.192. 15865943

[9] Wattel E , Preudhomme C , Hecquet B , Vanrumbeke M , Quesnel B , Dervite I , Morel P , Fenaux P . P53 mutations are associated with resistance to chemotherapy and short survival in hematologic malignancies. Blood. 1994; 84:3148–57. 10.1182/blood.v84.9.3148.3148. 7949187

[10] Reles A , Wen WH , Schmider A , Gee C , Runnebaum IB , Kilian U , Jones LA , El-Naggar A , Minguillon C , Schönborn I , Reich O , Kreienberg R , Lichtenegger W , Press MF . Correlation of p53 mutations with resistance to platinum-based chemotherapy and shortened survival in ovarian cancer. Clin Cancer Res. 2001; 7:2984–2997. 11595686

[11] Mogi A , Kuwano H . TP53 mutations in nonsmall cell lung cancer. J Biomed Biotechnol. 2011; 2011:583929. 10.1155/2011/583929. 21331359 PMC3035360

[12] Mitsudomi T , Hamajima N , Ogawa M , Takahashi T . Prognostic significance of p53 alterations in patients with non-small cell lung cancer: a meta-analysis. Clin Cancer Res. 2000; 6:4055–4063. 11051256

[13] Steels E , Paesmans M , Berghmans T , Branle F , Lemaitre F , Mascaux C , Meert AP , Vallot F , Lafitte JJ , Sculier JP . Role of p53 as a prognostic factor for survival in lung cancer: a systematic review of the literature with a meta-analysis. Eur Respir J. 2001; 18:705–719. 10.1183/09031936.01.00062201. 11716177

[14] Ahrendt SA , Hu Y , Buta M , McDermott MP , Benoit N , Yang SC , Wu L , Sidransky D . P53 mutations and survival in stage I non-small-cell lung cancer: results of a prospective study. J Natl Cancer Inst. 2003; 95:961–70. 10.1093/jnci/95.13.961. 12837832

[15] Döhner H , Stilgenbauer S , Benner A , Leupolt E , Kröber A , Bullinger L , Döhner K , Bentz M , Lichter P . Genomic Aberrations and Survival in Chronic Lymphocytic Leukemia. N Engl J Med. 2000; 343:1910–1916. 10.1056/NEJM200012283432602. 11136261

[16] Poeta ML , Manola J , Goldwasser MA , Forastiere A , Benoit N , Califano JA , Ridge JA , Goodwin J , Kenady D , Saunders J , Westra W , Sidransky D , Koch WM . TP53 mutations and survival in squamous-cell carcinoma of the head and neck. N Engl J Med. 2007; 357:2552–61. 10.1056/NEJMoa073770. 18094376 PMC2263014

[17] Joerger AC , Fersht AR . Structure-function-rescue: the diverse nature of common p53 cancer mutants. Oncogene. 2007; 26:2226–42. 10.1038/sj.onc.1210291. 17401432

[18] Canale M , Petracci E , Delmonte A , Chiadini E , Dazzi C , Papi M , Capelli L , Casanova C , De Luigi N , Mariotti M , Gamboni A , Chiari R , Bennati C , et al. Impact of TP53 mutations on outcome in EGFR-mutated patients treated with first-line Tyrosine Kinase Inhibitors. Clin Cancer Res. 2017; 23:2195–2202. 10.1158/1078-0432.CCR-16-0966. 27780855

[19] Yang JC , Sequist LV , Geater SL , Tsai CM , Mok TS , Schuler M , Yamamoto N , Yu CJ , Ou SH , Zhou C , Massey D , Zazulina V , Wu YL . Clinical activity of afatinib in patients with advanced non-small-cell lung cancer harbouring uncommon EGFR mutations: a combined post-hoc analysis of LUX-Lung 2, LUX-Lung 3, and LUX-Lung 6. Lancet Oncol. 2015; 16:830–838. 10.1016/S1470-2045(15)00026-1. 26051236

[20] VanderLaan PA , Rangachari D , Mockus SM , Spotlow V , Reddi HV , Malcolm J , Huberman MS , Joseph LJ , Kobayashi SS , Costa DB . Mutations in TP53, PIK3CA, PTEN and other genes in EGFR mutated lung cancers: Correlation with clinical outcomes. Lung Cancer. 2017; 106:17–21. 10.1016/j.lungcan.2017.01.011. 28285689 PMC5351777

[21] Seidel D , Zander T , Heukamp LC , Peifer M , Bos M , Fernández-Cuesta L , Leenders F , Lu X , Ansén S , Gardizi M , Nguyen C , Berg J , Russell P , et al. A genomics-based classification of human lung tumors. The Clinical Lung Cancer Genome Project (CLCGP) and Network Genomic Medicine (NGM). Sci Transl Med. 2013; 5:209ra153. 10.1126/scitranslmed.3006802. 24174329 PMC4006630

[22] Yu HA , Jordan E , Ni A , Feldman D , Rodriguez C , Kim HR , Kris MG , Solit DB , Berger MF , Ladanyi M , Arcila ME , Riely GJ . Concurrent genetic alterations identified by next-generation sequencing in pre-treatment, metastatic EGFR-mutant lung cancers. J Clin Oncol. 2016 (Suppl 15); 34:9053 10.1200/jco.2016.34.15_suppl.9053.

[23] Kron A , Alidousty C , Scheffler M , Merkelbach-Bruse S , Seidel D , Riedel R , Ihle MA , Michels S , Nogova L , Fassunke J , Heydt C , Kron F , Ueckeroth F , et al. Impact of TP53 mutation status on systemic treatment outcome in ALK-rearranged non-small-cell lung cancer. Ann Oncol. 2018; 29:2068–2075. 10.1093/annonc/mdy333. 30165392 PMC6225899

[24] Oxnard GR , Arcila ME , Sima CS , Riely GJ , Chmielecki J , Kris MG , Pao W , Ladanyi M , Miller VA . Acquired resistance to EGFR tyrosine kinase inhibitors in EGFR-mutant lung cancer: distinct natural history of patients with tumors harboring the T790M mutation. Clin Cancer Res. 2011; 17:1616–22. 10.1158/1078-0432.CCR-10-2692. 21135146 PMC3060283

[25] Oldenhuis CN , Oosting SF , Gietema JA , de Vries EG . Prognostic versus predictive value of biomarkers in oncology. Eur J Cancer. 2008; 44:946–953. 10.1016/j.ejca.2008.03.006. 18396036

[26] Seike M , Inoue A , Sugawara S , Morita S , Hosomi Y , Ikeda S , Watanabe K , Takahashi K , Fujita Y , Harada T , Minato K , Takamura K , Kobayashi K , Nukiwa T . 1382PD Phase III study of gefitinib (G) versus gefitinib+carboplatin+pemetrexed (GCP) as first-line treatment for patients (pts) with advanced non-small cell lung cancer (NSCLC) with EGFR mutations (NEJ009). Ann Oncol. 2018 (Suppl 8); 29 10.1093/annonc/mdy292.005.

[27] Synnott NC , Murray A , McGowan PM , Kiely M , Kiely PA , O'Donovan N , O'Connor DP , Gallagher WM , Crown J , Duffy MJ . Mutant p53: a novel target for the treatment of patients with triple-negative breast cancer? Int J Cancer. 2017; 140:234–246. 10.1002/ijc.30425. 27615392

[28] Demir S , Boldrin E , Sun Q , Hampp S , Tausch E , Eckert C , Ebinger M , Handgretinger R , Kronnie GT , Wiesmüller L , Stilgenbauer S , Selivanova G , Debatin KM , Meyer LH . Therapeutic targeting of mutant p53 in pediatric acute lymphoblastic leukemia. Haematologica. 2020; 105:170–181. 10.3324/haematol.2018.199364. 31073076 PMC6939517

