# Supplementary material for: TP53 co-mutations in EGFR mutated patients in NSCLC stage IV: A strong predictive factor of ORR, PFS and OS in EGFR mt+ NSCLC
Source: Oncotarget. 2020 Jan 21;11(3):250–64. doi: 10.18632/oncotarget.27430 (PMC6980625; doi:10.18632/oncotarget.27430)
Supplement: Supplementary file 1 [file oncotarget-11-250-s001.pdf]

TP53 co-mutations in EGFR mutated patients in NSCLC stage IV: A strong predictive factor of ORR, PFS and OS in EGFR mt+ NSCLC

SUPPLEMENTARY MATERIALS

PATIENTS AND METHODS

Microtome sections (5 µM) were prepared from FFPE-tissue of NSCLC samples and one object slide was HE stained for tumor evaluation by a pathologist. Tumor tissue was gained from the remaining slides by manual microdissection, or in case material was limited, enriched by Laser Capture Microdissection (Leica CTR6500). DNA extraction was performed either manually (Macharey Nagel) or semi-automated (Maxwell MDx, Promega). Against the background of technological advances in recent years, EGFR exon 18–21 and TP53 (exons 5–9) mutational analysis were performed by direct Sanger sequencing, Cobas® test or NGS-based methods. Alternatively, an amplicon-based NGS panel (Illumina platform) was used to detect mutations in 17 relevant genes, including TP53. Part of the samples were analyzed with a hybrid capture based target enrichment followed by massively parallel sequencing (Hybrid Capture NGS, NeoSelect, NEO New Oncology). The library preparation for the samples was performed using the Agilent SureSelect XT Kit as per the manufacturers' recommendations.

In an effort to specify the functional significance of the respective mutations in further detail, we included additional parameters in order to modify differentiation into pathogenic vs. non-pathogenic TP53 mutations. These mutations are likely to interfere with TP53 function significantly. Also, if an Align-GVGD score of C65 was reached, mutations were classified as pathogenic. Specifically, DNA-contact-mutations R273C, R273G, R248Q were reclassified as pathogenic mutations, since functional impairment is likely [2]. Mutation R280I is located within the LSH2- (loop-sheet-helix region 2), which is part of the DNA-binding core and was therefore re-categorized as pathogenic. Mutations H179R and C176S constitute Zn^{2+}-binding sites and were therefore also regarded as pathogenic upon review. In addition, online protein prediction programs were used to estimate the pathogenicity of all other p53 missense changes located inside the key DNA-binding domain of p53 L1 (codons 112–141), L2 (codons 163–195), L3 (codons 236–251), LSH1 (119–135) and LSH2 (272–287).

A third classification was recently proposed by the group of Canale et al. [3]. The authors characterized a cohort of EGFR mt+ patients that in 30.1% of cases carried additional TP53 mutations and these were categorized based on exons. TP53 mutations within exon 8 were associated with significantly lower DCR, and shorter PFS and overall survival [3].

REFERENCES

1. Poeta ML, Manola J, Goldwasser MA, Forastiere A, Benoit N, Califano JA, Ridge JA, Goodwin J, Kenady D, Saunders J, Westra W, Sidransky D, Koch WM. TP53 mutations and survival in squamous-cell carcinoma of the head and neck. N Engl J Med. 2007; 357:2552–61. https://doi.org/10.1056/NEJMoa073770. [PubMed]
2. Joerger AC, Fersht AR. Structure-function-rescue: the diverse nature of common p53 cancer mutants. Oncogene. 2007; 26:2226–42. https://doi.org/10.1038/sj.onc.1210291. [PubMed]
3. Canale M, Petracci E, Delmonte A, Chiadini E, Dazzi C, Papi M, Capelli L, Casanova C, De Luigi N, Mariotti M, Gamboni A, Chiari R, Bennati C, et al. Impact of TP53 mutations on outcome in EGFR-mutated patients treated with first-line Tyrosine Kinase Inhibitors. Clin Cancer Res. 2017; 23:2195–2202. https://doi.org/10.1158/1078-0432.CCR-16-0966. [PubMed]

Supplementary Table 1: Baseline characteristics of patients with EGFR mutations (*n* = 75)

	Exon 19 *n* = 42	Exon 21 *n* = 27	Exon 18/20 (I/III) *n* = 6	*p*-value	All *n* = 75
Age (years)					
mean	63.6 (45–85)	66.5 (45–81)	72 (60–85)	0.08	65.4 (45–85)
median	64	70	74	0.06	66
Sex					
men	15 (35.7%)	8 (29.6%)	0	0.20	23 (30.7%)
women	27 (64.3%)	19 (70.4%)	6 (100%)		52 (69.3%)
in total	42 (100%)	27 (100%)	6 (100%)		75 (100%)
Smoking status				0.21	
never/light	26 (62%)	22 (81.5%)	3 (50%)		51 (68%)
ex/current	13 (31%)	5 (18.5%)	3 (50%)		21 (36%)
not known	3 (7%)	0	0		3 (4%)
in total	42 (100%)	27 (100%)	6 (100%)		75 (100%)
ECOG				0.01	
0	24 (57.1%)	16 (59.3%)	3 (50%)		43 (57.3%)
1	16 (38.1%)	7 (25.9%)	0		23 (30.7%)
2	2 (4.8%)	3 (11.1%)	3 (50%)		8 (10.7%)
3	0	1 (3.7%)	0		1 (1.3%)
in total	42 (100%)	27 (100%)	6 (100%)		75 (100%)
CCI				0.13	
0–1	12 (28.6%)	5 (18.5%)	0		17 (22.7%)
2–3	20 (47.6%)	16 (59.3%)	2 (33.3%)		38 (50.6%)
>3	10 (23.8%)	6 (22.2%)	4 (67.7%)		20 (26.6%)
in total	42 (100%)	27 (100%)	6 (100%)		75 (100%)
CNS metastasis					
CNS primary				0.55	
• yes	8 (19%)	8 (29.6%)	1 (16.7%)		17 (22.7%)
• no	34 (81%)	19 (70.4%)	5 (83.3%)		58 (77.3%)
in total	42 (100%)	27 (100%)	6 (100%)		75 (100%)
CNS PD				0.38	
• yes	12 (28.6%)	4 (14.8%)	1 (16.7%)		17 (22.7%)
• no	30 (71.4%)	23 (85.2%)	5 (83.3%)		58 (77.3%)
in total	42 (100%)	27 (100%)	6 (100%)		75 (100%)
TP53 mutation classifications					
Poeta et al. [1]				0.94	
- non-disruptive	9 (21.4%)	4 (14.8%)	0		13 (17.4%)
- disruptive	8 (19.1%)	6 (22.2%)	2 (33.3%)		16 (21.3%)
- WT	16 (38.1%)	12 (44.5%)	2 (33.3%)		30 (40%)
- not done	9 (21.4%)	5 (18.5)	2 (33.4%)		16 (21.3%)
- in total	42 (100%)	27 (100%)	6 (100%)		75 (100%)
TP53 Structural/ biophysical classification				0.95	
- non-pathogenic	4 (9.5%)	2 (7.4%)	1 (16.7%)		7 (9.4%)
- pathogenic	13 (31%)	8 (29.6%)	1 (16.7%)		22 (29.3%)
- WT	16 (38.1%)	12 (44.5%)	2 (33.3%)		30 (40%)
- not done	9 (21.4%)	5 (18.5%)	2 (33.3%)		16 (21.3%)
- in total	42 (100%)	27 (100%)	6 (100%)		75 (100%)
TP53 exon 8 vs. non-exon 8 (18)				0.83	
- exon 8	5 (11.9%)	1 (3.7%)	0		6 (8%)
- non-exon 8	12 (28.6%)	9 (33.3%)	2 (33.3%)		23 (30.7%)
- WT	16 (38.1%)	12 (44.5%)	2 (33.3%)		30 (40%)
- not done	9 (21.4%)	5 (18.5%)	2 (33.4%)		16 (21.3%)
- in total	42 (100%)	27 (100%)	6 (100%)		75 (100%)

Supplementary Table 2: Resistance mutations/amplifications of EGFR mutated patients

TP53 Poeta et al. classification [1]

	EGFR T790M mutation	EGFR other resistance mutations	EGFR amplifications	Met gene amplifications	All resistance mutations/ amplifications
non-disruptive TP53 (n = 13)	4 (20%)	0	0	0	4 (14.8%)
disruptive TP53 (n = 16)	6 (30%)	1 (33.3%)	2 (66.7%)	0	9 (33.3%)
TP53 WT (n = 29)	9 (45%)	2 (66.7%)	1 (33.3%)	1 (100%)	13 (48.2%)
TP53 not done (n = 16)	1 (5%)	0	0	0	1 (3.7%)
ALL (n = 74)	20 (100%)	3 (100%)	3 (100%)	1 (100%)	27 (100%)

	Odds Ratio T790M	*p*-value
non-disruptive vs. disruptive	0.74 [0.16, 3.50]	0.70
non-disruptive vs. TP53 WT	0.99 [0.24, 4.07]	0.99
disruptive vs. TP53 WT	1.33 [0.37, 4.80]	0.66

TP53 structural/biophysical classification

	EGFR T790M mutation	EGFR other resistance mutations	EGFR amplifications	Met gene amplifications	All resistance mutations/ amplifications
non-pathogenic (n = 7)	2 (10%)	0	0	0	2 (7.4%)
pathogenic (n = 22)	8 (40%)	1 (33.3%)	2 (66.7%)	0	11 (40.7%)
TP53 WT (n = 29)	9 (45%)	2 (66.7%)	1 (33.3%)	1 (100%)	13 (48.2%)
TP53 not done (n = 16)	1 (5%)	0	0	0	1 (3.7%)
ALL (n = 74)	20 (100%)	3 (100%)	3 (100%)	1 (100%)	27 (100%)

	Odds ratio T790M	*p*-value
non-pathogenic vs. pathogenic	0.70 [0.11, 4.48]	0.71
non-pathogenic vs. TP53 WT	0.89 [0.14, 5.48]	0.90
pathogenic vs. TP53 WT	1.27 [0.39, 4.10]	0.69

TP53 exon 8 vs. non-exon 8 classification [3]

	EGFR T790M mutation	EGFR other resistance mutations	EGFR amplifications	Met gene amplifications	All Resistance mutations/ amplifications
exon 8 (n = 6)	1 (5%)	0	0	0	1 (3.7%)
non-exon 8 (n = 23)	9 (45%)	1 (33.3%)	2 (66.7%)	0	12 (44.4%)
TP53 WT (n = 29)	9 (45%)	2 (66.7%)	1 (33.3%)	1 (100%)	13 (48.2%)
TP53 not done (n = 16)	1 (5%)	0	0	0	1 (3.7%)
ALL (n = 74)	20 (100%)	3 (100%)	3 (100%)	1 (100%)	27 (100%)

	Odds Ratio T790M	*p*-value
exon 8 vs. non-exon 8	0.39 [0.04, 4.06]	0.43
exon 8 vs. TP53 WT	0.56 [0.05, 5.70]	0.62
non-exon 8 vs. TP53 WT	1.43 [0.45, 4.51]	0.54

Supplementary Table 3: Results of the multivariate logistic regression

TP53 Poeta et al. classification				
**Objective response**				
**Variable**	**OR**	**95% CI**	***p*-value**	**Reference category**
TP53 non-disruptive/disruptive mutation	0.03	0.00–0.56	0.019	TP53 WT
EGFR uncommon mutation	0.04	0.00–0.88	0.041	EGFR common mutation
ECOG 2	0.00	0.00–0.20	0.005	ECOG 0 &1
Initial CNS metastasis<br>• no	0.03	0.00–0.95	0.047	Initial CNS metastasis<br>• yes
**TP53 structural/biopysical classification**				
**Objective response**				
**Variable**	**OR**	**95% CI**	***p*-value**	**Reference category**
TP53 pathogenic mutation	0.12	0.02–0.75	0.024	TP53 non-pathogenic
EGFR uncommon mutation	0.03	0.00–0.54	0.017	EGFR common mutation
ECOG 2	0.01	0.00–0.27	0.005	ECOG 0 &1
Initial CNS metastasis<br>• no	0.04	0.00–0.82	0.037	Initial CNS metastases<br>• yes
**TP53 Poeta et al. classification**				
**Progression free survival**				
**Variable**	**HR**	**95% CI**	***p*-value**	**Reference category**
TP53 nondisruptive/disruptive mutation	3.07	1.45–6.50	0.003	TP53 WT
ECOG 2	4.23	1.56–11.50	0.005	ECOG 0 &1
**TP53 structural/biopysicall classification**				
**Progression free survival**				
**Variable**	**HR**	**95% CI**	***p*-value**	**Reference category**
TP53 pathogenic mutation	6.19	2.80–13.70	<0.001	TP53 non-pathogenic
ECOG 2	4.12	1.51–11.29	0.006	ECOG 0 & 1
EGFR uncommon mutation	3.19	1.21–8.43	0.019	EGFR common status
**TP53 Poeta et al. classification**				
**Overall survival**				
**Variable**	**HR**	**95% CI**	***p*-value**	**Reference category**
TP53 nondisruptive/disruptive mutation	4.08	1.87–8.88	<0.001	TP53 WT
ECOG 2	6.48	2.21–18.97	0.001	ECOG 0 & 1
Initial CNS metastasis<br>• no	0.36	0.15–0.84	0.019	Initital CNS metastasis<br>• yes
**TP53 structural/biophysical classification**				
**Overall survival**				
**Variable**	**HR**	**95% CI**	***p*-value**	**Reference category**
TP53 pathogenic mutation	4.88	2.26–10.55	<0.001	TP53 non-pathogenic
ECOG 2	5.93	2.04–17.23	0.001	ECOG 0 & 1
Initital CNS metastasis<br>• no	0.35	0.15–0.81	0.014	Initital CNS metastasis<br>• yes
